# VaxGO: an interactive web tool for systems vaccinology data analysis

**DOI:** 10.1093/bioadv/vbaf101

**Published:** 2025-05-02

**Authors:** Wasim Aluísio Prates-Syed, Aline Aparecida Lima, Nelson Cortes, Evelyn Carvalho, Jaqueline Dinis Queiroz Silva, Bárbara Hamaguchi, Ricardo Durães-Carvalho, Otávio Cabral-Marques, Thomas Hagan, José E Krieger, Gustavo Cabral-Miranda

**Affiliations:** Institute of Tropical Medicine, Faculty of Medicine of the University of São Paulo, São Paulo, 05403-000, Brazil; Biomedical Sciences Institute, University of São Paulo, São Paulo, 05508-000, Brazil; Pro-Vaccine Union, University of São Paulo, Ribeirão Preto, São Paulo, 14040-900, Brazil; Institute of Tropical Medicine, Faculty of Medicine of the University of São Paulo, São Paulo, 05403-000, Brazil; Biomedical Sciences Institute, University of São Paulo, São Paulo, 05508-000, Brazil; Institute of Tropical Medicine, Faculty of Medicine of the University of São Paulo, São Paulo, 05403-000, Brazil; Biomedical Sciences Institute, University of São Paulo, São Paulo, 05508-000, Brazil; Institute of Tropical Medicine, Faculty of Medicine of the University of São Paulo, São Paulo, 05403-000, Brazil; Biomedical Sciences Institute, University of São Paulo, São Paulo, 05508-000, Brazil; Institute of Tropical Medicine, Faculty of Medicine of the University of São Paulo, São Paulo, 05403-000, Brazil; Biomedical Sciences Institute, University of São Paulo, São Paulo, 05508-000, Brazil; Institute of Tropical Medicine, Faculty of Medicine of the University of São Paulo, São Paulo, 05403-000, Brazil; Department of Microbiology, Immunology and Parasitology, São Paulo School of Medicine, Federal University of São Paulo, São Paulo, 04023-062, Brazil; Department of Morphology and Genetics, Federal University of São Paulo, São Paulo, 04021-001, Brazil; Biomedical Sciences Institute, University of São Paulo, São Paulo, 05508-000, Brazil; Department of Medicine, Division of Molecular Medicine, University of São Paulo School of Medicine, São Paulo, 01246-903, Brazil; Cincinnati Children's Hospital, Cincinnati, OH, 45229, United States; Heart Institute, Clinical Hospital, Faculty of Medicine, University of São Paulo, São Paulo, 05403-900, Brazil; Laboratory of Genetics and Molecular Cardiology, Clinical Hospital, Faculty of Medicine, University of São Paulo, 05403-900, Brazil; Institute of Tropical Medicine, Faculty of Medicine of the University of São Paulo, São Paulo, 05403-000, Brazil; Heart Institute, Clinical Hospital, Faculty of Medicine, University of São Paulo, São Paulo, 05403-900, Brazil; Laboratory of Genetics and Molecular Cardiology, Clinical Hospital, Faculty of Medicine, University of São Paulo, 05403-900, Brazil

## Abstract

**Motivation:**

RNA sequencing is crucial for investigating transcriptional patterns in immunology and vaccine research. However, the analysis of RNA sequencing data often requires programming skills, which can limit accessibility for researchers lacking such expertise.

**Results:**

We present VaxGO, an intuitive web-based tool designed to facilitate the analysis of differentially expressed genes in the context of immune processes and cells during vaccination. This tool integrates data from Gene Ontology, CellMarker 2.0, the MSigDB Vax collection, and other key studies, including transcriptional atlases of vaccines against COVID-19 and other diseases. VaxGO is an interactive, web-based tool, offering a user-friendly platform for exploring immune responses and vaccine efficacy without programming expertise.

**Availability and implementation:**

The VaxGO tool is available at https://github.com/wapsyed/VaxGO.

## 1 Introduction

RNA sequencing (RNAseq) has been widely used to describe transcriptional patterns in immunological applications, including immunization. Various studies have been conducted to understand immune responses in blood samples, which are easily obtainable and well-established for immunological analyses (Pulendran *et al.* 2011, Pulendran 2014, Raeven *et al.* 2019). In addition, a variety of tools were developed to assess cell populations and immunological signatures. A well-established tool is CellMarker, which helps to identify cell populations using curated genetic markers (Hu *et al.* 2023). However, cellular markers alone do not provide in-depth descriptions of cellular and systemic processes. For this, biological processes described in Gene Ontology are often used (Ashburner *et al.* 2000, Aleksander *et al.* 2023), and include manually curated and bioinformatics inferenced annotations. Nevertheless, these processes are not easily categorized at an immunological level in a way that is easy to comprehend. An important breakthrough was the development of blood transcription modules (BTM), which outlined transcriptional modules associated with patterns found in blood in multiple studies (Li *et al.* 2014). In addition, MSigDB also integrated a multitude of gene expression data in different contexts, including cancer and immunology (Mootha *et al.* 2003, Subramanian *et al.* 2005). These tools together provide valuable insights into immunological omics, including those related to vaccination.

To better understand specifically vaccination, two atlases have been developed. Hagan *et al.* (2022) described the transcriptional patterns of 13 different vaccines under various conditions. Subsequently, Prates-Syed *et al.* (2024) applied this framework to analyze vaccines used against COVID-19. Given the extensive diversity of gene sets, it is crucial to develop a tool that facilitates efficient data analysis for novel vaccine experiments. Such a tool would enable comparative assessments and assists in evaluating the advancement of vaccine development stages.

We introduce VaxGO, a comprehensive repository of gene datasets, carefully curated to advance the understanding of immune processes, cell markers, and vaccination responses.

## 2 Methods

### 2.1 Gene sets retrieval and manual annotation

**Figure 1.**
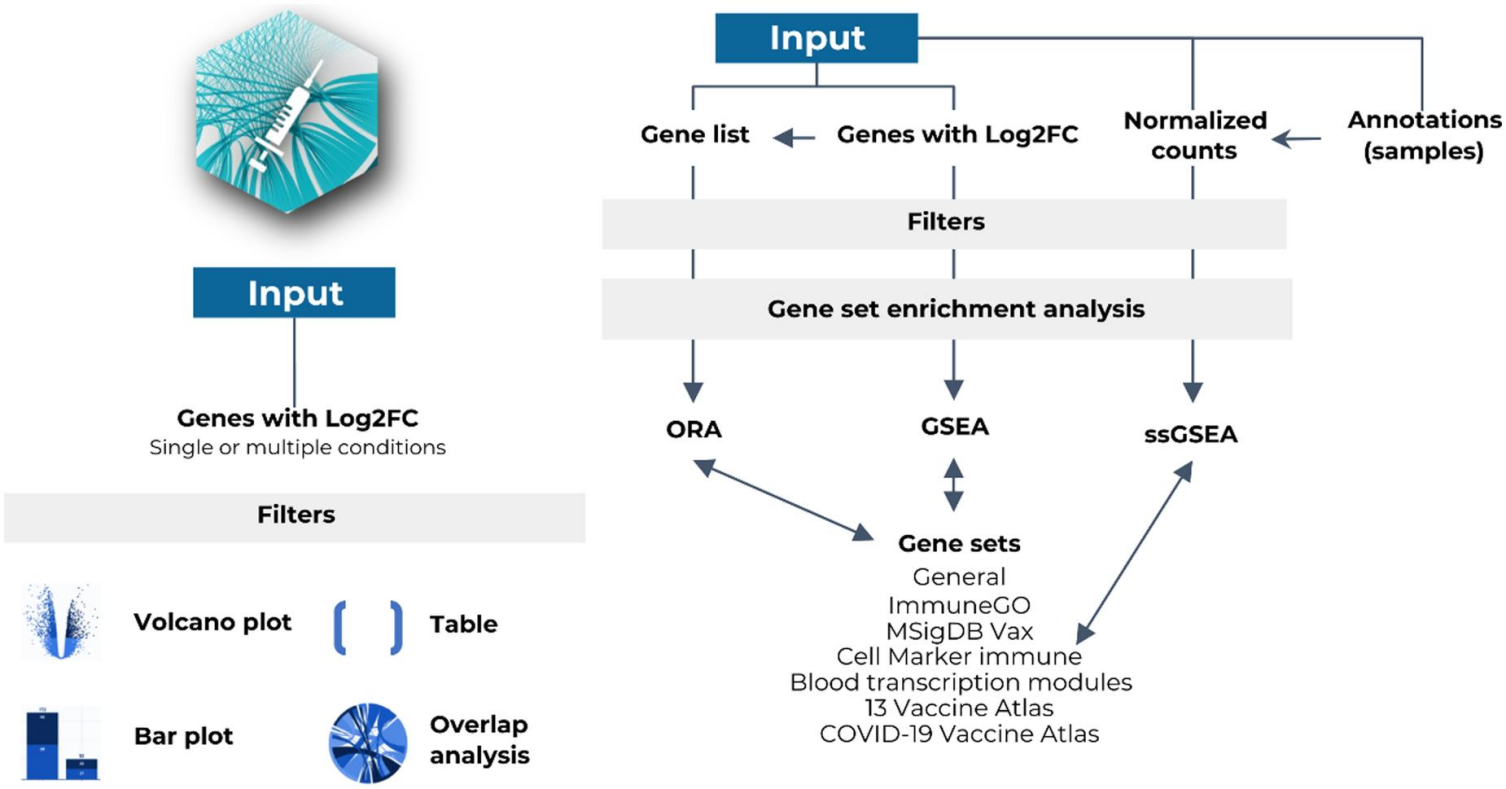
Overview of the VaxGO tool. VaxGO provides interactive interfaces for visualizing DEGs through volcano plots, tables, and bar plots. It also enables overlap analysis of DEGs across different conditions when multiple conditions are provided. Additionally, VaxGO facilitates GSEA, over-representation analysis (ORA), and ssGSEA, using integrated databases such as ImmuneGO, MSigDB VAX, CellMarker, BTM, and vaccine atlases.

We compiled gene sets from different sources, including databases and articles, and annotated them manually according to immunological and vaccinology knowledge (Fig. 1). The Gene Ontology data were retrieved using the GO.db R package (Carlson 2019) to build an immunological gene set database. To refine the dataset, we filtered the biological processes related to immune function, specifically the ‘immune system process’ (GO: 0002376). The resulting dataset was named ImmuneGO. To facilitate subsequent analyses, biological processes associated with the immune system were categorized into three distinct groups: ‘immune system,’ ‘immune subsystem,’ and when related to specific cells, classified under the ‘immune cell’ category. We focused on a classical division of processes involved in vaccination, encompassing the complement system and both adaptive and innate immune systems. This includes inflammation; antiviral response and interferon production; neutrophils; mast cells; monocytes; antigen receptors, capture, processing; and presentation by antigen-presenting cells of innate response (monocytes, macrophages, and dendritic cells) and adaptive response (B cells and T cells). In addition, we considered the interaction of these components with T cells, a critical component that acts as an effector, stimulator, and regulator of adaptive cellular and humoral responses. It also included genes related to antimicrobial response and to nonspecific leukocytes. To simplify a top–bottom analysis, from gene sets to individual genes, gene sets were unified representing processes involved in immunological subsystems and cells of the innate response. These included ‘inflammation,’ ‘antigen receptors, capture, processing, and presentation,’ ‘interferon and antiviral responses,’ ‘macrophages,’ ‘dendritic cells,’ ‘monocytes,’ ‘neutrophils,’ ‘eosinophils,’ ‘mast cells,’ and ‘NK cells.’ In addition, we grouped the gene sets related to the adaptive response, such as ‘cellular adaptive immune system,’ ‘humoral adaptive immune system,’ ‘Ig-mediated response,’ ‘T cells,’ and ‘B cells,’ as well as other immunological processes, such as ‘complement immune system,’ ‘antimicrobial response,’ and ‘general.’ Cellular and humoral adaptive immune system gene sets comprise all processes related to these responses, except for respective organ development and cellular processes, including differentiation, chemotaxis, homeostasis, and proliferation.

To perform a comparative analysis involving vaccines targeting different pathogens across different platforms, we used the MSigDB VAX subset and two atlases (Liberzon *et al.* 2015). This collection comprises gene sets that have been positively and negatively regulated, and are associated with human vaccines, encompassing a diverse range of platforms and pathogenic targets. It is important to note that this dataset did not include vaccines specifically against corona viruses. Subsequently, gene sets were enriched with various attributes through manual annotation derived from the available information on the descriptive pages of the dataset. These gene sets cover a variety of vaccine-related conditions, including gene expression profiles before and after vaccination, comparisons between vaccinated and control groups, analyses of differentially expressed genes (DEGs) between groups immunized with different vaccines, samples from individuals administered booster doses, vaccinated individuals with and without adjuvants, varied doses, individuals with different levels of immune response, comparisons of DEGs between these groups, and other comparisons. An additional category was created to include gene sets related to correlations with antibody titers, cells, adverse events, age, protection, and antimicrobial activity. A category was also included for gene sets called ‘SUBSET,’ comprising genes resulting from gene signature analyses, top DEGs in different vaccination conditions, and genes used in machine learning algorithm training sets. Finally, a category was created for the gene sets related to individuals challenged with the target pathogen. We also used datasets from other atlases. DEGs from COVID-19 vaccines were downloaded from the GitHub repository (Prates-Syed *et al.* 2024) [https://github.com/wapsyed/covidvax_atlas]. In addition, we integrated data from 13 vaccines targeting other diseases (Hagan *et al.* 2022), which was obtained from Immune Signatures Data Resource (Diray-Arce *et al.* 2021).

For enrichment of specific immune cell signatures in blood samples, we retrieved the CellMarker dataset and filtered human blood markers of leukocytes (Hu *et al.* 2023) [http://117.50.127.228/CellMarker/CellMarker_download.html]. The BTM dataset was downloaded from the supplementary files of the original publication (Li *et al.* 2014). Subsequently, these gene sets were manually grouped according to their phenotypes, following the same framework used on ImmuneGO, of which only immune transcription modules were filtered. We also provide a grouped dataset with ImmuneGO and BTM for filtering and annotating immune genes, expanding the coverage of gene annotation.

### 2.2 Enrichment analysis

For gene overlap analysis, we used the vegan package (Oksanen *et al.* 2025) to calculate the Jaccard distances, and base R to perform Fisher's exact test. Gene set enrichment analysis (GSEA) and single-sample GSEA (ssGSEA) were performed using the Clusterprofiler (Yu *et al.* 2012, Wu *et al.* 2021) and corto (Mercatelli *et al.* 2020), respectively.

### 2.3 Data visualization and interaction

For data visualization, we used the R packages ggplot2, plotly, and circlize (Gu *et al.* 2014). To create the web application, we used the R packages—Shiny (Chang *et al.* 2024) and Flexdashboards (Aden-Buie *et al.* 2023) in RStudio (RStudio Team 2022) and deployed it at a server on shinyapps.io.

## 3 Results

### 3.1 Coverage of immune processes and cell profiling

**Figure 2.**
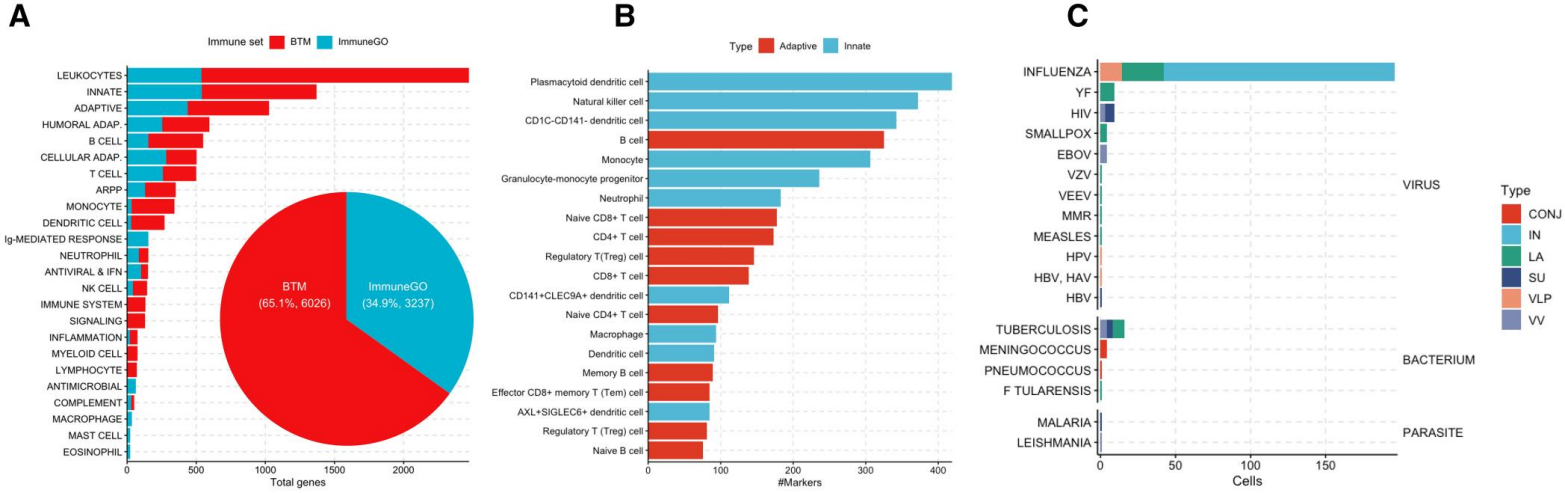
Analysis of immune processes, cell markers, and vaccines in public databases included in VaxGO. (A) Coverage of immune processes categorized by ImmuneGO and BTM, highlighting the percentage distribution of pathways related to innate immunity and adaptive immunity. (B) Annotation of cell markers for human leukocyte populations, emphasizing the distribution of markers for innate immune cells and adaptive immune cells. (C) Integration of vaccine-specific gene sets, including viral, bacterial, and parasitic vaccines.

Integration of BTM and ImmuneGO significantly expanded coverage of immune processes, comprising exclusive genes for each dataset, with 6026 and 3237 genes, respectively. Key innate immune processes, such as interferon response, antigen processing and presentation, and inflammation, dominated the dataset, while adaptive immune processes, including B cell and T cell-related, were also well-represented (Fig. 2A). VaxGO incorporated CellMarker PBMC, a manually curated database of human leukocyte markers (Fig. 2B). Dendritic cells exhibited the highest marker diversity, followed by monocytes and T cells. This granularity enables precise identification of cell populations in blood transcriptomic data.

### 3.2 Vaccine-specific gene set integration

The MSigDB Vax collection enabled comparative analysis of vaccines targeting viral, bacterial, and parasitic pathogens (Fig. 2C). However, it is limited to predefined sets of upregulated and downregulated genes without associated log2-fold change values or *P* values, restricting its applicability to over-representation analysis and GSEA analyses. In contrast, the integration of the 13 vaccine atlas and the COVID-19 atlas, which include log-fold change values and *P* values, allows for more advanced analyses, such as correlation analysis. This integration unifies diverse vaccine technologies and conditions, enabling comprehensive cross-vaccine comparisons. Vaccine-specific DEGs were annotated with key metadata, including adjuvant use, dosage regimens, and correlations with antibody titers, providing a more nuanced understanding of immune responses across different vaccine platforms (Hagan *et al.* 2022, Prates-Syed *et al.* 2024).

### 3.3 User-friendly interface for immune transcriptomics analysis of human vaccination

**Figure 3.**
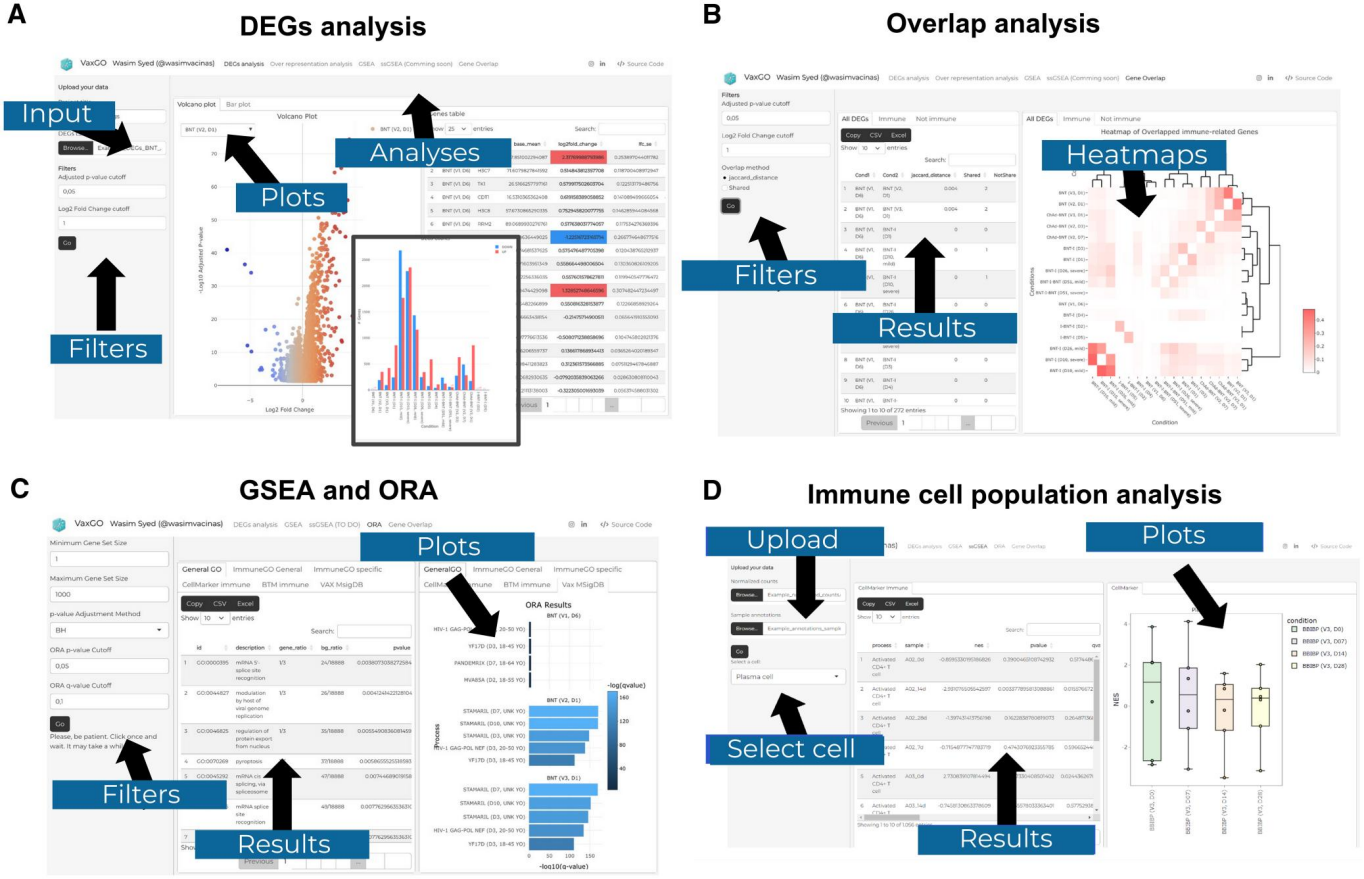
Web tool flowchart demonstration. (A) On the homepage, users can upload a comma-separated values (CSV) file containing differential gene expression (DEGs) across single or multiple conditions. The file should include log2-fold change (L2FC) values, P values, and adjusted P values (p.adjust). Users can filter the L2FC and p.adjust values and visualize DEGs through an interactive volcano plot and view summaries of upregulated and downregulated genes in a bar chart. (B) The tool also enables comprehensive overlap analysis of DEGs across multiple conditions, incorporating statistical assessments such as Fisher's exact test and Jaccard distance calculations. Additionally, it supports correlation analysis using both Pearson and Spearman methods. Results are presented in an interactive heatmap for easy comparison. (C) Subsequent pages allow users to perform over-representation analysis (ORA), GSEA, and ssGSEA (D) using a comprehensive collection of immune and vaccination-related gene sets. With the Vax MSigDB collection, the user can also filter the study type and subtype, as well as the type of vaccine, pathogen, and other categories.

VaxGO provides an interactive, user-friendly platform for analyzing immune-related transcriptomic data (Fig. 3). Users can upload differential gene expression data in comma-separated values format, including log2-fold change, *P* values, and adjusted *P* values. The tool generates an interactive volcano plot for visualizing upregulated and downregulated genes based on statistical significance thresholds. Users can dynamically filter genes and export the processed data for further analysis.

Comparative analyses across multiple conditions are enabled via Fisher's exact test, Jaccard distance, and correlation analysis, enabling the identification of shared and unique DEGs across multiple conditions. These comparisons are visualized as interactive heatmaps, facilitating the exploration of gene expression patterns. Subsequent modules allow functional enrichment analysis through over-representation analysis, GSEA, and ssGSEA, using integrated immune and vaccine-specific gene sets.

## 4 Discussion

VaxGO addresses a critical gap in systems vaccinology by democratizing access to advanced transcriptomic analysis for nonprogrammers. It features a manually curated immune gene set called ImmuneGO, which categorizes immune-related biological processes; CellMarker PBMC, which organizes human leukocyte markers; and the MSigDB Vax collection for comparative vaccine analysis. Additionally, it includes data derived from the COVID-19 Vaccination Atlas (Prates-Syed *et al.* 2024, GitHub), the 13 vaccine atlas (Hagan *et al.* 2022), and BTM (Li *et al.* 2014). The inclusion of vaccine-specific datasets and immune annotations from Gene Ontology and experiments—ImmuneGO and BTM, respectively—allows researchers to benchmark novel vaccines against established platforms, identifying conserved or unique immune signatures. Developed with Shiny in R, VaxGO offers an interactive web-based interface, allowing users to explore and analyze immune responses from immunization and other applications without programming skills. Together, these resources offer a robust foundation for analyzing DEGs related to the immune system, advancing our understanding of immune responses and vaccine development.

Although the tool is particularly useful for the analysis of the immune system response and comparison between vaccines, we strongly recommend the ShinyGO tool and others for general analysis of nonimmune genes (Ge *et al.* 2020). Additionally, its reliance on precurated databases may limit flexibility for novel gene sets. Future updates could incorporate machine learning to predict unannotated immune pathways or integrate single-cell RNAseq data for higher resolution.

In conclusion, the modular design and interactive visualizations presented by our tool helps researchers to unravel complex immune mechanisms, enabling the translation of omics data into insights for new vaccines.

## Data Availability

The source code, integrated databases, and both the browser-based and downloadable versions of the tool are available on GitHub at https://github.com/wapsyed/VaxGO.
